# Canaloplasty: A Minimally Invasive and Maximally Effective Glaucoma Treatment

**DOI:** 10.1155/2015/485065

**Published:** 2015-10-01

**Authors:** Mahmoud A. Khaimi

**Affiliations:** Dean McGee Eye Institute, University of Oklahoma, Oklahoma City, OK 73104, USA

## Abstract

Canaloplasty is a highly effective, minimally invasive, surgical technique indicated for the treatment of open-angle glaucoma that works by restoring the function of the eye's natural outflow system. The procedure's excellent safety profile and long-term efficacy make it a viable option for the majority of glaucoma patient types. It can be used in conjunction with existing drug based glaucoma treatments, after laser or other types of incisional surgery, and does not preclude or affect the outcome of future surgery. Numerous scientific studies have shown Canaloplasty to be safe and effective in lowering IOP whilst reducing medication dependence. A recent refinement of Canaloplasty, known as ab-interno Canaloplasty (ABiC), maintains the IOP-lowering and safety benefits of traditional (ab-externo) Canaloplasty using a more efficient, simplified surgical approach. This paper presents a review of Canaloplasty indications, clinical data, and complications, as well as comparisons with traditional incisional glaucoma techniques. It also addresses the early clinical evidence for ABiC.

## 1. Introduction

Over a decade after the first Canaloplasty procedure was performed, evidence pertaining to the long-term efficacy and safety of this noninvasive, restorative glaucoma surgery continues to accumulate. Today, more than 50 peer-reviewed clinical studies attest to the fact that Canaloplasty is as effective as trabeculectomy in lowering intraocular pressure (IOP) and reducing dependence on medications [1–6].

The advantages of Canaloplasty are multiple. It is a minimally invasive, nonpenetrating procedure that does not create a permanent fistula in the wall of the eye and does not require a bleb, hence avoiding the potential spectrum of bleb-related complications associated with traditional glaucoma surgery [7]. Such a minimally invasive surgical approach not only dispenses with bleb-related complications but critically provides long-term reduction in IOP, reduces the need for glaucoma medications [1], and is a good option for those patients with open-angle glaucoma (OAG) who are not yet ready for more invasive traditional surgeries such as trabeculectomy or tube shunts.

Having evolved from viscocanalostomy [8], Canaloplasty successfully addresses problems associated with that earlier procedure, such as recollapse of Schlemm's canal and closure of the ostia, by enhancing the natural outflow in three main ways [9]: firstly, transtrabecular flow is enhanced in part by tensioning the meshwork and opening up the trabecular plates; secondly, circumferential viscodilation of Schlemm's canal provides IOP lowering; and, finally, viscodilation of Schlemm's canal also opens up the collector channels. The creation of a scleral lake and a Descemet's window provides an additional insurance, ensuring sustained IOP reduction over the long-term.

By addressing all of the possible sites of resistance, including potentially occluded collector channels, Canaloplasty enables surgeons to obtain postoperative pressures in the range of low-to-mid teens, similar to that achieved with trabeculectomy.

## 2. Surgical Procedure

Canaloplasty surgery begins with the creation of a conjunctival flap, usually in the supranasal quadrant to spare superior conjunctiva for possible future procedures, and a superficial scleral flap which is dissected forward into clear cornea [10]. The surgeon then sculpts a deep scleral flap and Schlemm's canal is opened. The deep scleral flap is removed and the two ostia of the canal are dilated with viscoelastic. A microcatheter (iTrack 250, Ellex iScience, Inc., Freemont, CA, USA) is then inserted and guided within Schlemm's canal for the entire 360 degrees until it emerges at the other end of the canal opening. A stent suture is then tied to the catheter's distal tip and the microcatheter is reversed back through Schlemm's canal in the opposite direction. Inward distension of the trabecular meshwork is achieved by knotting the suture under tension. The superficial scleral flap is then repositioned and can be sutured to ensure a watertight closure. The conjunctival flap is also returned to its original position and sutured in a watertight fashion.

Viscodilation is a fundamental component of the procedure. Circumferential (360°) catheterization of Schlemm's canal with the iTrack 250A, combined with gentle viscodilation, breaks adhesions within Schlemm's canal, stretches the trabecular plates creating microperforations within the inner wall of the trabecular meshwork thus allowing flow into Schlemm's canal, and separates herniations of the inner wall of the trabecular meshwork into the outer wall collector channels.

One of the more compelling reasons for using Canaloplasty is that it takes due account of the eye's natural outflow system and restores the physiological outflow pathways. This is in contrast to most other glaucoma treatments which not only fail to address the eye's natural drainage system but may also, in some cases, even impede this outflow function.

It is important to note that Canaloplasty, via both ab-externo and ab-interno approaches, is the only glaucoma treatment which addresses the collector channels. Studies undertaken in human and bovine POAG eyes, by Gong et al., have shown that the collector channels play a key role in blocking aqueous outflow in POAG eyes. Specifically, when inner wall tissue of the trabecular meshwork herniates into the collector channels, it blocks aqueous outflow [11, 12]. In POAG eyes fixed at 0 mmHg (*N* = 5), 73 collector channel ostia regions were examined, with 51 showing herniations (70%). In POAG eyes fixed at 10 mmHg (*N* = 2), 22 collector channel ostia regions were examined, with 21 showing herniations (95%). In contrast, in normal eyes (fixed at 0 mmHg), 53 collector channel ostia regions were examined and 8 herniations were found (15%). A significant difference was found between normal and POAG eyes fixed at 0 mmHg (*p* = 0.0008).

## 3. Patient Selection

Canaloplasty is indicated for various forms of primary open-angle glaucoma, pseudoexfoliative glaucoma [13], and pigmentary glaucoma [14]. Pediatric patients with congenital glaucoma have also benefited from Canaloplasty [15], and the procedure has been shown to be both safe and effective in patients undergoing cataract surgery or contact lens wearers [16]. It can also be successfully performed in patients with failed trabeculectomy in which Schlemm's canal has been left undamaged from previous filtrating surgeries [17].

Other potential candidates for Canaloplasty include patients at high risk for infection or bleeding and those with enhanced wound healing. Canaloplasty offers an effective alternative for these patients as it aims to restore the physiologic outflow pathways independent of external wound healing [18]. Canaloplasty may also be a better option for patients with active lifestyles who experience difficulty adhering to the rigorous postoperative care required after trabeculectomy.

Patients who will not benefit from Canaloplasty include those with angle-closure glaucoma, narrow-angle glaucoma (not undergoing concurrent lens extraction), neovascular glaucoma, and posttraumatic glaucoma, and in eyes with interruption or damage to Schlemm's canal due to previous ocular surgery or extensive thermal laser trabeculoplasty with peripheral anterior synechiae [10].

## 4. The Clinical Evidence

One of the first landmark trials for Canaloplasty was a multicenter prospective trial carried out at 15 clinical sites in the United States of America, Great Britain, and Germany in 2005 [1]. This groundbreaking study included 157 eyes of 157 OAG patients with a historical pressure of 21 mmHg or higher, with many of them on maximum tolerated medical therapy. Canaloplasty procedures were carried out on 121 eyes while 36 eyes underwent phacocanaloplasty, that is, Canaloplasty combined with cataract extraction.

The published three-year data from that trial validated the potential benefits of Canaloplasty, demonstrating a significant and sustained IOP reduction and reduced need for medications in adult patients with OAG. It also confirmed the excellent short- and long-term safety profile of the procedure.

Looking at the results in more detail, of the 89 procedures performed with successful placement of a suture, there was a 34% mean decrease in IOP from baseline (23.5 mmHg ± 4.5 to 15.5 mmHg ± 3.5) and a 53% mean reduction in postoperative medications (1.9 ± 0.8 to 0.9 ± 0.9) at three-year follow-up. When phacoemulsification was combined with Canaloplasty and successful suture placement, 27 eyes had a 42% mean decrease in IOP (23.5 mmHg ± 5.2 to 13.6 mmHg ± 3.6) and an 80% mean reduction of postoperative medications (1.5 ± 1 to 0.3 ± 0.5). While transient hyphema was the most common side effect, occurring in 10.2% of eyes, a study by Grieshaber et al. has shown that hyphema can, in fact, be considered to be a sign of successful reconnection with the ocular venous system and therefore of good prognosis [19, 20]. Sustained hypotony and related complications, however, did not occur.

Such positive experiences are also increasingly reflected in the scientific literature, with the outcomes in several recently published studies equaling or surpassing those of the 2005 trial. In 2011, Grieshaber et al. published the results of a prospective study of 32 patients with OAG in which the mean IOP fell from 27.3 ± 5.6 mmHg preoperatively to 12.8 ± 1.5 mmHg at 12 months and 13.1 ± 1.2 mmHg at 18 months [3]. A more recent study by Brusini of 214 eyes from 185 OAG patients with a maximum of four-year follow-up reported a mean IOP reduction of 42.2% [10].

## 5. Safety Profile and Complications

While the IOP-lowering benefits of Canaloplasty and trabeculectomy are similar, the safety profiles of the two techniques are vastly different. In the absence of a subconjunctival bleb, Canaloplasty offers significantly fewer postsurgical complications and a simplified follow-up compared to trabeculectomy [7]. The vast majority of patients tend to have a perfectly normal looking eye after a few weeks, without any ocular discomfort.

Potential intraoperative complications associated with Canaloplasty include inability to cannulate Schlemm's canal, Descemet membrane detachment, and improper microcatheter passage [1, 2, 12, 21, 22]. The most frequent postoperative complications associated with Canaloplasty include hyphema or microhyphema, cataract formation, IOP spikes, and hypotony [12].

Compared to trabeculectomy complications, many of these problems are easily resolved and some should perhaps not be classified as complications at all. As noted earlier, a study by Grieshaber showed that the absence of microhyphaema on the first postoperative day actually seems to be a negative prognostic indicator in uneventful Canaloplasty procedures in patients with primary open-angle glaucoma (POAG) [18].

Another recent study by Jaramillo et al. recorded an incidence of Descemet membrane detachment after Canaloplasty of 7.4% [22], while other studies report a rate between 1.6% and 9.1% [16] or even lower [23]. While choroidal detachment is sometimes included in the list of potential complications associated with Canaloplasty, its occurrence is very rare indeed. A comprehensive review by Harvey and Khaimi in 2011 [16] found that no choroidal detachment, suprachoroidal haemorrhage, blebitis, or bleb-associated endophthalmitis had been reported in the scientific literature.

Fewer postoperative complications equate to happier patients. A recent quality of life study found that Canaloplasty patients were more satisfied with their surgery than their trabeculectomy counterparts [24]. The 176 Canaloplasty patients were happier and less stressed concerning the surgery than the 152 trabeculectomy patients (84% versus 51%).

## 6. Ab-Interno Canaloplasty: A Natural Evolution

Evolving directly from Canaloplasty, ab-interno Canaloplasty (ABiC) is a new MIGS procedure that may achieve similar IOP-lowering effects to traditional (ab-externo) Canaloplasty in patients with mild-to-moderate POAG.

As with traditional Canaloplasty, ABiC is designed to access, catheterize, and viscodilate all aspects of outflow resistance—the trabecular meshwork, Schlemm's canal, and the distal outflow system beginning with the collector channels. The key difference, however, is that no tensioning suture is required to maintain the IOP reduction with the ab-interno approach and the procedure spares conjunctival manipulation for future procedures if required.

Like traditional Canaloplasty, ABiC addresses all the key structures that control ocular outflow—the trabecular meshwork, Schlemm's canal, and collector channels. It also follows the same dilatation principles as traditional Canaloplasty where gentle application of viscoelastic during insertion allows the compressed tissue planes of trabecular meshwork and sclera to separate and any herniated trabecular meshwork tissue to withdraw from collector channels. Again, similar to traditional Canaloplasty, after circumferential passage of the iTrack 250A Canaloplasty microcatheter, viscoelastic (Healon or Healon GV, Abbott Medical Optics) is emitted upon single clicks of the viscoinjector knob.

## 7. Other MIGS Approaches

Other MIGS lower IOP by addressing specific—but not all—aspects of the ocular outflow system. The trabectome uses an electrosurgical pulse to ablate the trabecular meshwork and inner wall of Schlemm's canal, while the iStent works as a trabecular microbypass by allowing aqueous humor to flow directly from the anterior chamber into Schlemm's canal, thus circumventing the trabecular meshwork [25]. Another device, the Hydrus, an 8 mm long device, is inserted into Schlemm's canal to improve ocular outflow from the anterior chamber to Schlemm's canal by acting as an intracanalicular scaffold [26]. The CyPass Suprachoroidal Microstent, an investigational MIGS, facilitates outflow from the anterior chamber to the suprachoroidal space, while the Aquesys Subconjunctival Implant (also an investigational MIGS device) is placed into the subconjunctival space to create a filtering bleb [26].

In addition to addressing all aspects of ocular outflow in one procedure, ABiC is very efficient and less invasive than other MIGS. It retains all the benefits of traditional Canaloplasty in terms of IOP reduction and minimal complications while offering a simplified surgical approach.

## 8. Clinical Considerations

As ABiC is recommended early in the disease process, the primary indication for ABiC is patients with mild-to-moderate glaucoma on medical therapy. However, it may also be considered a first-line option or in patients who have undergone laser trabeculoplasty and for patients noncompliant to glaucoma medications. Patients with exfoliative glaucoma and those in whom glaucoma surgery in the fellow eye has failed may also be considered for ABiC. Exclusion criteria are similar to traditional Canaloplasty and should not be performed in patients with neovascular glaucoma, chronic angle-closure, angle recession/peripheral anterior synechiae, or narrow-angle glaucoma. ABiC is most frequently performed in conjunction with phacoemulsification; however, it is not limited to a combination procedure and may be performed alone.

## 9. Summary

Canaloplasty is very effective in lowering IOP, has an excellent safety profile, and can be used to treat a wide variety of glaucoma types. The evidence in the scientific literature attests to the fact that this technique really is minimally invasive and maximally effective in treating mild-to-moderate POAG. Furthermore, the clinical evidence indicates that ABiC, a new minimally invasive glaucoma treatment, is safe and effective in mild-to-moderate POAG with similar IOP-lowering effects to traditional (ab-externo) Canaloplasty. Unlike other MIGS, ABiC ensures that all potential “blockages” in the ocular outflow pathway are addressed, including distal structures such as the collector channels which have been shown to play a key role in blocking aqueous outflow in POAG eyes, and may thereby potentially offer better clinical outcomes.
